# Studies of Artificial Intelligence/Machine Learning Registered on ClinicalTrials.gov: Cross-Sectional Study With Temporal Trends, 2010-2023

**DOI:** 10.2196/57750

**Published:** 2024-10-25

**Authors:** Shoko Maru, Michael D Matthias, Ryohei Kuwatsuru, Ross J Simpson Jr

**Affiliations:** 1 Graduate School of Medicine Juntendo University Tokyo Japan; 2 Clinical Study Support Inc Nagoya Japan; 3 Matthias IT Pty Ltd Brisbane Australia; 4 Department of Radiology School of Medicine Juntendo University Tokyo Japan; 5 Division of Cardiology School of Medicine University of North Carolina Chapel Hill Chapel Hill, NC United States

**Keywords:** artificial intelligence, machine learning, deep learning, trends, health care, cross-sectional study, health disparities, data-source disparities, publication bias, registry, ClinicalTrials.gov

## Abstract

**Background:**

The rapid growth of research in artificial intelligence (AI) and machine learning (ML) continues. However, it is unclear whether this growth reflects an increase in desirable study attributes or merely perpetuates the same issues previously raised in the literature.

**Objective:**

This study aims to evaluate temporal trends in AI/ML studies over time and identify variations that are not apparent from aggregated totals at a single point in time.

**Methods:**

We identified AI/ML studies registered on ClinicalTrials.gov with start dates between January 1, 2010, and December 31, 2023. Studies were included if AI/ML-specific terms appeared in the official title, detailed description, brief summary, intervention, primary outcome, or sponsors’ keywords. Studies registered as systematic reviews and meta-analyses were excluded. We reported trends in AI/ML studies over time, along with study characteristics that were fast-growing and those that remained unchanged during 2010-2023.

**Results:**

Of 3106 AI/ML studies, only 7.6% (n=235) were regulated by the US Food and Drug Administration. The most common study characteristics were randomized (56.2%; 670/1193; interventional) and prospective (58.9%; 1126/1913; observational) designs; a focus on diagnosis (28.2%; 335/1190) and treatment (24.4%; 290/1190); hospital/clinic (44.2%; 1373/3106) or academic (28%; 869/3106) sponsorship; and neoplasm (12.9%; 420/3245), nervous system (12.2%; 395/3245), cardiovascular (11.1%; 356/3245) or pathological conditions (10%; 325/3245; multiple counts per study possible). Enrollment data were skewed to the right: maximum 13,977,257; mean 16,962 (SD 288,155); median 255 (IQR 80-1000). The most common size category was 101-1000 (44.8%; 1372/3061; excluding withdrawn or missing), but large studies (n>1000) represented 24.1% (738/3061) of all studies: 29% (551/1898) of observational studies and 16.1% (187/1163) of trials. Study locations were predominantly in high-income countries (75.3%; 2340/3106), followed by upper-middle-income (21.7%; 675/3106), lower-middle-income (2.8%; 88/3106), and low-income countries (0.1%; 3/3106). The fastest-growing characteristics over time were high-income countries (location); Europe, Asia, and North America (location); diagnosis and treatment (primary purpose); hospital/clinic and academia (lead sponsor); randomized and prospective designs; and the 1-100 and 101-1000 size categories. Only 5.6% (47/842) of completed studies had results available on ClinicalTrials.gov, and this pattern persisted. Over time, there was an increase in not only the number of newly initiated studies, but also the number of completed studies without posted results.

**Conclusions:**

Much of the rapid growth in AI/ML studies comes from high-income countries in high-resource settings, albeit with a modest increase in upper-middle-income countries (mostly China). Lower-middle-income or low-income countries remain poorly represented. The increase in randomized or prospective designs, along with 738 large studies (n>1000), mostly ongoing, may indicate that enough studies are shifting from an in silico evaluation stage toward a prospective comparative evaluation stage. However, the ongoing limited availability of basic results on ClinicalTrials.gov contrasts with this field’s rapid advancements and the public registry’s role in reducing publication and outcome reporting biases.

## Introduction

The number of studies on artificial intelligence (AI) and machine learning (ML) registered on ClinicalTrials.gov has significantly increased since 2016 [1,2]. While the rapid growth of research on AI/ML is apparent [1-3], the downsides in this field have also come to light. These include the lack of level I/II evidence as robust proof of the clinical and economic impacts of AI [4], lack of clinically meaningful outcomes beyond measures of technical accuracy [5], lack of uniformity and standardization in AI reporting [6,7], incomplete and poor reporting [8-10], limited publication of AI/ML results [11,12], or data-source disparities with minority subgroups potentially disadvantaged by factors such as race, gender, and socioeconomic background [13,14].

Previous cross-sectional studies using ClinicalTrials.gov as of December 2020 [2] and March 2022 [1] showed that the number of AI/ML studies continued to grow rapidly. However, it is unclear whether this growth in number reflects an increase in desirable study attributes or merely perpetuates the same issues previously raised in the literature. For example, are study designs conducive to level I/II evidence increasing over time? Do AI/ML studies continue to run predominantly in advanced economies? While research using ClinicalTrials.gov often reports country-specific distributions [1,2], examining the global distribution by economic measures could further elucidate whether technological power is concentrated in a few high-income countries.

We aimed to evaluate temporal trends over the past 14 years, identifying variations that may not be immediately apparent from aggregated totals at a single point in time. We highlighted the fastest-growing characteristics over time and those that have remained unchanged. We also characterized AI/ML studies by the following: *who* (lead sponsor sector), *what* (clinical specialty, AI/ML terms used in study descriptions), *when* (start year, primary completion year, time to results posting), *where* (study location, geographic and economic), *why* (primary study purpose), and *how* (study design, enrollment).

## Methods

### Data Source

We conducted a cross-sectional analysis using ClinicalTrials.gov, a trial registry and results database maintained by the US National Library of Medicine. We sourced data from the Clinical Trials Transformation Initiative Aggregate Analysis of ClinicalTrials.gov (CTTI AACT) [15], which allows open access to the complete set of trials registered in ClinicalTrials.gov, including additional fields that are not readily available in direct exports from ClinicalTrials.gov. The CTTI AACT data dictionary is publicly accessible [16]. A static version of the CTTI AACT database was downloaded for analysis on February 6, 2024, via PostgreSQL (pSQL). The pSQL codes used are provided in Multimedia Appendix 1.

### Study Selection

We used text-based search to identify relevant AI/ML studies. Our search strategies were informed by systematic reviews published during 2019-2023 [9,17-20]. We used search terms related to AI/ML methodologies and specific model architectures as used in the literature. Terms are not necessarily mutually exclusive or hierarchical (eg, “multilayer perceptron” under “machine learning”). The search strategy is detailed in Table S1 in Multimedia Appendix 2, with corresponding SQL codes available in Multimedia Appendix 1.

### Inclusion Criteria (If All of the Following Met)

1. Start date: January 1, 2010, to December 31, 2023.
2. The following search terms were used:
  *artificial intelligence, ai-based, augmented intelligence, deep learning, convolutional neural network, deep neural network, artificial neural network, recurrent neural network, generative adversarial network, deep reinforcement learning, machine learning, bayesian network, classification tree, elastic net, gradient boosting, xgboost, k nearest neighbour, multilayer perceptron, support vector machine, natural language processing, naive bayes, random forest, regression tree, reinforcement learning, supervised learning, unsupervised learning*.
  These terms were searched for in the official title, brief summary, detailed descriptions, intervention, primary outcome, and sponsors’ keywords.

### Exclusion Criteria (If Any of the Following Met)

1. Studies registered as a meta-analysis, systematic review, scoping review, literature review, or protocol.
2. AI/ML-related terms were part of proper nouns (eg, organization, product, study name).
3. AI/ML-related terms were found, but they were unrelated to the study. For example, search terms were mentioned only in passing (eg, “previous research on AI showed promise,” “despite advances in machine learning”).

First, SQL was used to exclude studies containing any of the following terms: protocol*, design*, review*, systematic review*, scoping review*, literature review*, meta analys*s (* denotes a wildcard, equivalent to % in SQL codes). Second, the lead author selected studies with fewer than 5 search-word hits to confirm their relevance, and irrelevant studies were excluded (eg, those involving “neural networks” of a biological nature). Third, 2 reviewers independently examined study descriptions and assessed eligibility. Discrepancies were resolved through discussion and by revisiting details on ClinicalTrials.gov. Where study descriptions were unclear or insufficient, we opted to include studies unless they explicitly met the exclusion criteria, prioritizing sensitivity over specificity.

The study flow diagram is provided in Figure S1 in Multimedia Appendix 2. The pSQL codes used are provided in Multimedia Appendix 1.

### Data Extraction and Analysis

#### Overview

We extracted the following data fields from the CTTI AACT database: study start date, completion date, primary completion date, overall status, study type, number of sites, phase, enrollment, randomization status, masking, interventional model, primary purpose, target disease/condition, intervention, time perspective, lead sponsor, lead sponsor agency class, funding source, US Food and Drug Administration (FDA)–regulated status, sex, age, healthy volunteers, study location, and results availability.

Additionally, we created the 5 variables described in the following sections.

#### Enrollment—Categorical

Enrollment (continuous) was categorized as follows: 1-100; 101-1000; 1001-5000; 5001-10,000; 10,001-20,000; 20,001-30,000; and >30,000. We used the most recently updated enrollment data: planned or actual, whichever was more recent.

#### “Other” Sponsor Sectors—Disaggregated

As the sponsors of AI/ML research can be diverse, the “OTHER” and “OTHER_GOV” fields as classified in ClinicalTrials.gov are uninformative. We reclassified them into the following based on lead sponsor names: hospital/clinic, academia, industry, government, individual, research institute, nonprofit, and network. Academic hospitals were included in “hospital/clinic.”

#### Study Location—Global Distribution, Geographic and Economic

We counted studies geographically and by gross national income (GNI)–based classification as per the World Bank [21]. Each country was classified by 2022 GNI per capita: low income (US $1135 or less); lower middle income (US $1136 to US $4465); upper middle income (US $4466 to US $13,845); and high income (US $13,846 or more).

#### Clinical Specialty or Disease Area

Clinical specialties were assigned using the Medical Subject Headings (MeSH) largest headings. Since one registered study can involve substudies, resulting in multiple specialties, we assigned the MeSH heading with the highest count to each study identifier.

In cases where multiple MeSH headings had the same highest count (ties), all those terms were included (multiple MeSH headings per study identifier). If MeSH headings were missing, we used condition/disease terms submitted by sponsors to infer the clinical areas.

Note that “radiology” is a not an organ specific term and not a major MeSH heading directly linked to diseases. For example, specific disease imaging indexed under terms such as “breast neoplasms/radiography” or “lung diseases/radiography” had been preclassified as neoplasms or respiratory diseases. Screening procedures (eg, breast, colorectal) had been preclassified as neoplasms in the original data. Thus, compared to other study findings, radiology may be underrepresented, while neoplasms may be overrepresented.

#### Days From Primary Completion to Posting of Results

Among completed studies marked with “has results,” we calculated the time between “primary completion date” and “results first posted date.” We also assessed whether results were posted within 1 year after study completion (yes/no).

We used descriptive statistics and performed analyses in Microsoft Excel and Python (Python Software Foundation). We followed the Strengthening the Reporting of Observational Studies in Epidemiology (STROBE) guidelines [22]. Ethics approval was not required because only publicly available data were analyzed.

## Results

### Overview

The ClinicalTrials.gov search yielded 3378 records. Of those, 272 studies were excluded: not AI/ML-related (n=265); systematic reviews or meta-analyses (n=2); and the term “artificial intelligence” merely being a part of the organization, product, or study name (n=5). After excluding 272 studies, 3106 studies were included for analysis (Figure S1 in Multimedia Appendix 2).

Tables 1 and 2 show the study characteristics. Overall, the number of AI/ML studies increased rapidly after 2017, and 62.8% (1951/3106) started in the 2021 to 2023 period. Of the 3016 studies, 842 studies (27.1%) were completed, 1694 (54.5%) were active (not yet recruiting; recruiting; enrolling by invitation; active, not recruiting), 95 (3.1%) were stopped, and 475 (15.3%) were unknown. There were more observational studies (61.6%) than interventional studies (38.4%); more parallel assignments (89.2%) than crossover, factorial, or single assignments; more open-labeled studies (63%) than ones that used any form of masking; more single-center (77.2%) than multicenter (22.8%) studies; and more studies with 2 arms (56.8%) than 1 (34.3%) or 3 (9%) arms. The study phase was mostly “not applicable” (93%). Studies involving adults aged 18-65 years (58.1%) were most common, followed by adults and seniors aged 18 and above (27.5%). While some studies accepted healthy volunteers (35.2%), the majority did not (64.8%). Only 235 (7.6%) were FDA regulated.

**Table 1:** Characteristics of artificial intelligence/machine learning studies (N=3106) registered on ClinicalTrials.gov, 2010-2023^a^.

Study characteristics						Values			
**Start year (n=3106), n (%)**									
	2010				12 (0.4)				
	2011				19 (0.6)				
	2012				18 (0.6)				
	2013				22 (0.7)				
	2014				20 (0.6)				
	2015				42 (1.4)				
	2016				61 (2)				
	2017				81 (2.6)				
	2018				187 (6)				
	2019				255 (8.2)				
	2020				438 (14.1)				
	2021				599 (19.3)				
	2022				672 (21.6)				
	2023				680 (21.9)				
**Overall status (n=3106), n (%)**									
	Not yet recruiting				302 (9.7)				
	Recruiting				1116 (35.9)				
	Enrolling by invitation				90 (2.9)				
	Active, not recruiting				186 (6)				
	Completed				842 (27.1)				
	Withdrawn, terminated, suspended				95 (3.1)				
	Unknown status				475 (15.3)				
**Study type (n=3106), n (%)**									
	Interventional				1193 (38.4)				
	Observational				1668 (53.7)				
	Observational (patient registry)				245 (7.9)				
**Interventional model (n=742), n (%)**									
	Parallel assignment				662 (89.2)				
	Crossover assignment				58 (7.8)				
	Factorial assignment				22 (3)				
	Single assignment				0 (0)				
**Allocation (n=1193, interventional), n (%)**									
	Randomized				670 (56.2)				
	Nonrandomized				130 (10.9)				
	Not applicable/unknown				393 (32.9)				
**Masking (n=1193, interventional), n (%)**									
	Open-labeled				751 (63)				
	Single				232 (19.4)				
	Double				127 (10.6)				
	Triple or quadruple				83 (7)				
**Time perspective (n=1913, observational)^b^, n (%)**									
	Prospective				1126 (58.9)				
	Retrospective				430 (22.5)				
	Cross-sectional				210 (11)				
	Other				147 (7.7)				
**Number of facilities (n=2821), n (%)**									
	Single-center				2177 (77.2)				
	Multicenter				644 (22.8)				
**Number of study arms (n=1103), n (%)**									
	1				378 (34.3)				
	2				626 (56.8)				
	3				99 (9)				
**Phase (n=1193), n (%)**									
	Early phase 1				5 (0.4)				
	Phase 1				7 (0.6)				
	Phase 1/phase 2				6 (0.5)				
	Phase 2				28 (2.3)				
	Phase 2/phase 3				3 (0.3)				
	Phase 3				17 (1.4)				
	Phase 4				18 (1.5)				
	Not applicable				1109 (93)				
**Enrollment (n=3061)^c^**									
	Minimum				1				
	Maximum				13,977,257				
	Mean (SD)				16,962 (288,155)				
	Median (IQR)				255 (80-1000)				
**Enrollment (n=3061)^c^, n (%)**									
	**Interventional (n=1163)^c^**								
		1-100		497 (42.7)					
		101-1000		479 (41.2)					
		1001-5000		118 (10.1)					
		5001-10,000		25 (2.1)					
		10,001-20,000		8 (0.7)					
		20,001-30,000		7 (0.6)					
		>30,000		29 (2.5)					
		Withdrawn (n=0) or unknown		30					
	**All (n=3061)^c^**								
		1-100	951 (31.1)						
		101-1000	1372 (44.8)						
		1001-5000	449 (14.7)						
		5001-10,000	100 (3.3)						
		10,001-20,000	58 (1.9)						
		20,001-30,000	20 (0.7)						
		>30,000	111 (3.6)						
		Withdrawn (n=0) or unknown	45						
**FDA^d^ status (n=3106), n (%)**									
	FDA regulated (device or drug)				235 (7.6)				
	Not regulated				2871 (92.4)				
**Primary purpose (n=1190)^e^, n (%)**									
	Diagnostic				335 (28.2)				
	Treatment				290 (24.4)				
	Other				149 (12.5)				
	Prevention				97 (8.2)				
	Health services research				84 (7.1)				
	Screening				81 (6.8)				
	Supportive care				77 (6.5)				
	Basic science				57 (4.8)				
	Device feasibility				20 (1.7)				

^a^Tabulations exclude missing data, which may result in totals below 3106.

^b^Data on time perspective (eg, prospective) was only available for observational studies.

^c^Excludes withdrawn studies (n=0) and those with missing data.

^d^FDA: US Food and Drug Administration.

^e^Data on primary purpose was only available for interventional trials.

**Table 2:** Additional characteristics of artificial intelligence/machine learning studies (N=3106) registered on ClinicalTrials.gov, 2010-2023^a^.

Characteristics			Values
**Sex (n=3104), n (%)**			
	All	2863 (92.2)	
	Female	192 (6.2)	
	Male	49 (1.6)	
**Age (n=2896), n (%)**			
	Children (<18 y)	242 (8.4)	
	Adults (18-65 y)	1684 (58.1)	
	Elderly people (>65 y)	21 (0.7)	
	Children and adults	110 (3.8)	
	Adults and elderly people	796 (27.5)	
	All	43 (1.5)	
**Healthy volunteers (n=2854), n (%)**			
	Accepts healthy volunteers	1006 (35.2)	
	Does not accept healthy volunteers	1848 (64.8)	
**Lead sponsor sector (n=3106), n (%)**			
	Hospital/clinic	1373 (44.2)	
	Academia	869 (28)	
	Industry	407 (13.1)	
	Research institute	156 (5)	
	Individual	151 (4.9)	
	Government	71 (2.3)	
	Nonprofit	57 (1.8)	
	Network	9 (0.3)	
	Unknown	13 (0.4)	
**Funding source (n=3106), n (%)**			
	Industry	518 (16.7)	
	National Institutes of Health	143 (4.6)	
	Other	2445 (78.7)	
**Study location: region (n=2821), n (%)**			
	Europe	973 (34.5)	
	Asia/Pacific	841 (29.8)	
	North America	731 (25.9)	
	Middle East	84 (3)	
	Africa	37 (1.3)	
	Central and South America	31 (1.1)	
	Multiple regions	124 (4.4)	
**Study location: gross national income (n=3106)^b^, n (%)**			
	High income	2340 (75.3)	
	Upper middle income	675 (21.7)	
	Lower middle income	88 (2.8)	
	Low income	3 (0.1)	
**Study results (completed, n=842), n (%)**			
	Has results	47 (5.6)	
	No results	795 (94.4)	
**Study results (at least 1 year after primary completion date, n=691), n (%)**			
	Has results	45 (6.5)	
	No results	646 (93.5)	
**Study results (time in days from primary completion to result posting; n=47)**			
	Minimum	93	
	Maximum	1200	
	Median (IQR)	505 (399-676)	
**Clinical specialty (n=3245)^c^, n (%)**			
	Neoplasms (includes cancer screening)	420 (12.9)	
	Nervous system diseases	395 (12.2)	
	Cardiovascular diseases	356 (11)	
	Pathological conditions, signs and symptoms	325 (10)	
	Respiratory tract diseases	275 (8.5)	
	Digestive system diseases	253 (7.8)	
	Mental disorders	219 (6.7)	
	Endocrine, nutritional, or metabolic diseases	161 (5)	
	Female urogenital diseases and pregnancy complications	138 (4.3)	
	Skin and connective tissue diseases	89 (2.7)	
	Eye diseases	79 (2.4)	
	Musculoskeletal diseases	60 (1.8)	
	Behavior and behavior mechanisms	56 (1.7)	
	Male urogenital diseases	48 (1.5)	
	Surgical procedures, operative	38 (1.2)	
	Wounds and injuries	34 (1)	
	Chemically induced disorders	34 (1)	
	Bacterial infections and mycoses	33 (1)	
	Hemic and lymphatic diseases	31 (1)	
	Stomatognathic diseases	28 (0.9)	
	Radiology (not organ specific)	26 (0.8)	
	Otorhinolaryngologic diseases	22 (0.7)	
	Physiological phenomena	21 (0.6)	
	Immune system diseases	19 (0.6)	
	Congenital, hereditary, and neonatal diseases and abnormalities	18 (0.6)	
	Viral diseases	17 (0.5)	
	Critical care	14 (0.4)	
	Urology/nephrology	13 (0.4)	
	Geriatrics	11 (0.3)	
	Environment and public health	7 (0.2)	
	Circulatory and respiratory physiological phenomena	5 (0.2)	

^a^Tabulations exclude missing data, which may result in totals below 3106.

^b^Gross national income level grouping as per the World Bank.

^c^Multiple counts per study are possible and can exceed 3106.

### Study Design

**Figure 1:**
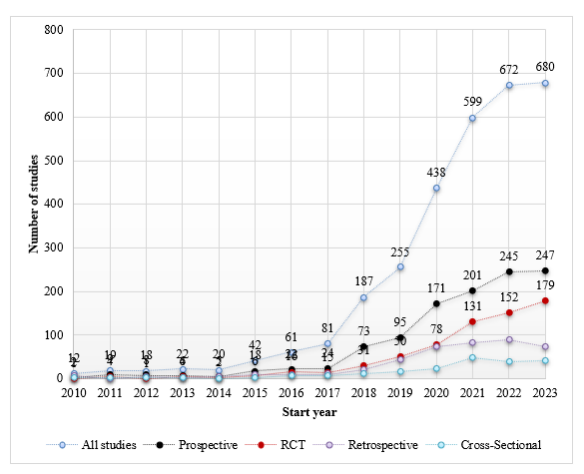
Study design characteristics by start year, 2010-2023. Excludes “unknown” or “other.” RCT: randomized controlled trial.

Among observational studies, a prospective design was more common (58.9%; 1126/1913) than retrospective (22.5%; 430/1913) or cross-sectional (11%; 210/1913) designs. Among trials, 56.2% (670/1193) were randomized, 10.9% (130/1193) were nonrandomized, and 32.8% (393/1193) were “not applicable” (Table 1). Figure 1 shows the numbers of all studies, including randomized controlled trials (RCTs) and prospective, retrospective, and cross-sectional studies, over time. From 2017 to 2023, the proportion of RCTs increased by 42.1%: from 18.52% (15/81) to 26.32% (179/680). The proportion of prospective studies increased by 22.6%: from 29.63% (24/81) to 36.32% (247/680). In contrast, the proportion of retrospective studies decreased by 11.9%, from 12.35% (10/81) to 10.88% (74/680), and cross-sectional studies declined by 28.5%, from 8.64% (7/81) to 6.18% (42/680).

### Primary Purpose

**Figure 2:**
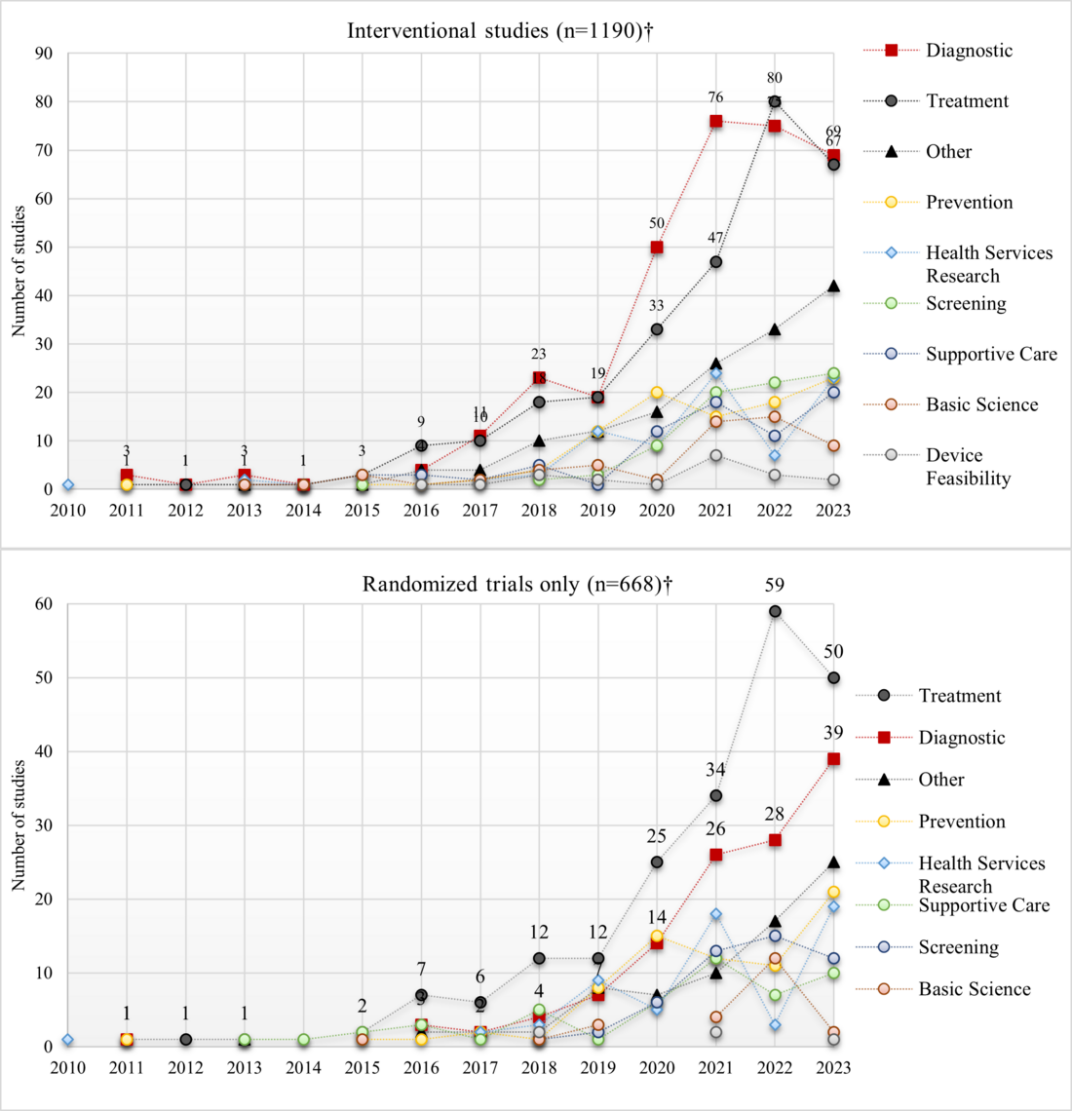
Primary study purposes by start year. Top: intervention studies; bottom: randomized trials, 2010-2023. †Excludes missing data.

Data on primary purpose were only available for interventional trials. Among the 1190 trials, the most common purposes were diagnostic (28.2%; n=335), treatment (24.4%; n=290), “other” (12.5%; n=149), and prevention (8.2%; n=97). However, the top 2 rankings were reversed when restricted to RCTs only: treatment (31.4%; n=210), diagnostic (18.6%; n=124), “other” (11.1%; n=74), and prevention (10.9%; n=73). Over time, studies focused on diagnosis and treatment increased faster than others (Figure 2).

### Clinical Specialty

The top 10 accounted for 81.1% (2631/3245) of all clinical specialties (Table 2). The 3245 specialties included neoplasms (12.9%; n=420); nervous system diseases (12.2%; n=395); cardiovascular diseases (11%; n=356); pathological conditions (10%; n=325); respiratory tract diseases (8.5%; n=275); digestive system diseases (7.8%; n=253); mental disorders (6.7%; n=219); endocrine, nutritional, or metabolic diseases (5%; n=161); female urogenital diseases and pregnancy complications (4.3%; n=138); and skin and connective tissue diseases (2.7%; n=89). Most of these started to increase during 2018-2020 (Figure S3 in Multimedia Appendix 2). See Table S3 in Multimedia Appendix 2 for the entire list of specialties over time.

### Lead Sponsor Sectors

**Figure 3:**
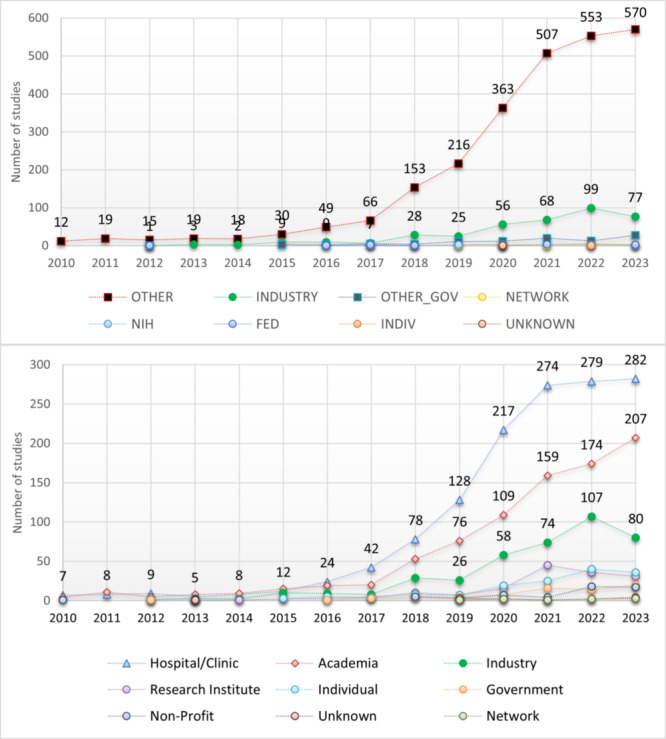
Lead sponsor sector by start year, 2010-2023. Top: as classified in ClinicalTrials.gov.; bottom: our reclassification based on sponsor names. NIH: National Institutes of Health.

We compared lead sponsor sectors classified in ClinicalTrials.gov versus our reclassification based on sponsor names (n=3106). In the original classification, the most common sector was “OTHER” (83.4%; n=2590), followed by “INDUSTRY” (12.4%; n=384) and “OTHER_GOV” (3.2%; n=98). However, our reclassification revealed the strong presence of hospitals/clinics (44.2%; n=1373) and academia (28%; n=869), followed by industry (13.1%; n=407) (Table 2; Figure S8 in Multimedia Appendix 2). Studies sponsored by hospitals/clinics, academia, and industry have increased since 2017 (Figure 3).

### Enrollment (Sample Size)

The enrollment data were skewed to the right: maximum 13,977,257; mean 16,962 (SD 288,155); and median 255 (IQR 80-1000). The most common size category overall was 101-1000 (44.8%). Among trials, 1-100 (42.7%; 497/1163) was most common, followed by 101-1000 (41.2%; 479/1163), whereas 101-1000 (46.7%; 893/1913) was most represented among observational studies (Table 1). This pattern did not materially change when stratified by sponsor sectors (Table S4 in Multimedia Appendix 2). Traditionally, the largest studies tend to be industry-sponsored [23], but this was not the case in our data. Among observational studies, the hospital/clinic sector, followed by academia, represented the highest number of studies in all size categories, including the largest (>30,000). Among trials, the hospital/clinic sector represented the most up to the 10,000 category. While interventional trials in the 1-100 and 101-1000 categories and observational studies in the 101-1000 category have increased the most over time (Figure S5 in Multimedia Appendix 2), 16.1% (187/1163) of trials and 29% (551/1898) of observational studies were large, with enrollments above 1000.

We also compared the overall enrollment distribution (1-100, 101-1000, and >1000) with that of prior reports that used the ClinicalTrials.gov data (Figure S6 in Multimedia Appendix 2). First, our data distribution was similar to that of previous research on AI studies up to March 2022 [1]. Second, when compared to 2 previous reports on non–disease-specific trials (non-AI/ML) during 2000-2019 and 2007-2010, the proportion of the largest category (>1000) was higher in our AI/ML trials (16.1%; 187/1163) than in the non-AI/ML trials: (3.8%; 5174/135,144 in 2000-2019 [23] and 3.9%; 1103/28,467 in 2007-2010 [24]). When restricted to RCTs alone, the proportion of the >1000 category was even higher in our AI/ML trials (19.8%; 129/653). Conversely, the proportion of the smallest category (1-100) was lower in our trials (42.7%; 497/1163) than in the non-AI/ML trials (63.2% [23] and 62.3% [24]).

### Sex-Specific Studies

The majority (92.2%; 2863/3104) was not sex-specific, followed by women-targeted (6.2%; 192/3104) and men-targeted (1.6%; 49/3104) studies (Table 2). Over time, AI/ML studies for women have increased since 2018 (Figure S7 in Multimedia Appendix 2).

### Study Location—Global Distribution, Geographic and Economic

The most common regions were Europe (34.5%; 973/2821), Asia/Pacific (29.8%; 841/2821), and North America (25.9%; 731/2821; Table 2), followed by the Middle East (3%; 84/2821). The most common countries were the United States (21.8%; 677/3106), China (17.7%; 550/3106), France (6.6%; 205/3106), the United Kingdom (6.2%; 193/3106), Italy (5.2%; 162/3106), and Taiwan (3.6%; 111/3106). See Table S2 in Multimedia Appendix 2 for a full list of countries, with the World Bank’s GNI-based distribution.

**Figure 4:**
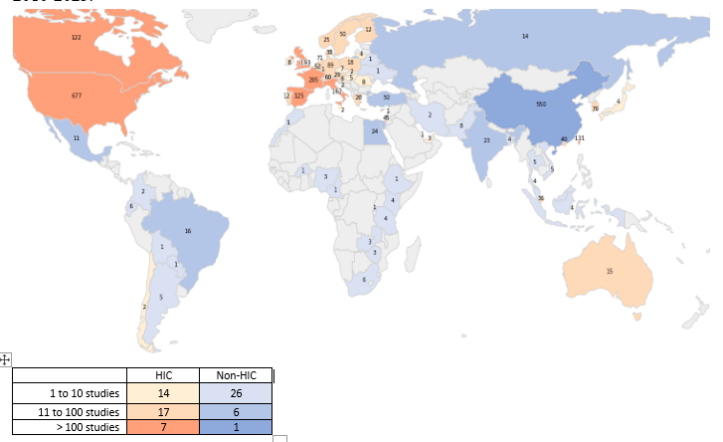
Study locations (geographic and economic) in artificial intelligence/machine learning studies, 2010-2023. Numbers denote the number of studies in each location. Studies with multiple locations are included, as well as single-country studies. Studies with unknown locations (missing) are not included in the counts. HIC: high-income country (as per the World Bank classification).

In Figure 4, 71 countries are color-divided into 38 high-income countries and 33 non–high-income countries. Most AI/ML studies were conducted in high-income countries (75.3%; 2340/3106), followed by upper-middle-income countries, including China (21.7%; 675/3106), lower-middle-income countries (2.8%; 88/3106), and low-income countries (0.1%; 3/3106) (Table 2). Studies in non–high-income countries have started to increase since 2020, particularly in Turkey (41 vs 50), Egypt (21 vs 24), India (15 vs 23), Brazil (15 vs 16), Russia (11 vs 14), and Mexico (8 vs 11), where the numbers in parentheses represent the totals for 2020-2023 compared to the totals for the entire period of 2010-2023.

**Figure 5:**
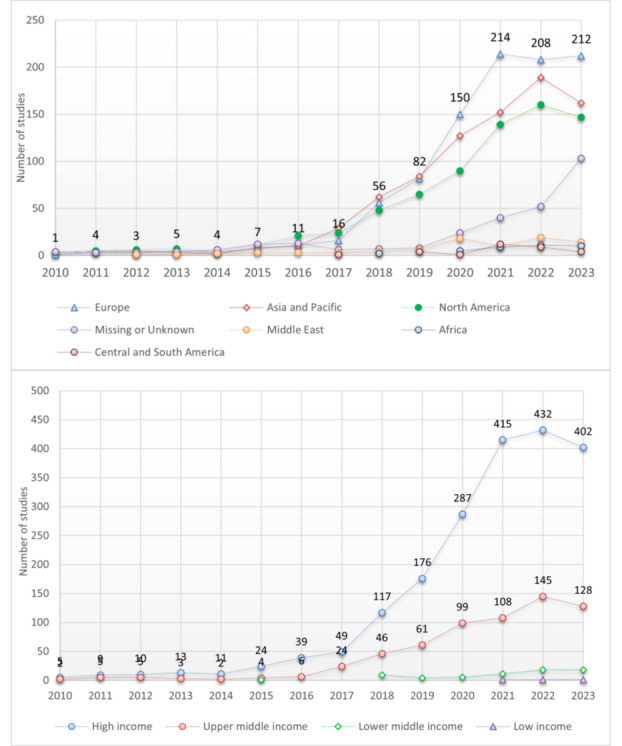
Study location by start year: (top) geographic and (bottom) economic. Single-country studies only (n=2696), 2010-2023.

As shown in Figure 5, studies in Europe, Asia/Pacific, and North America increased since 2018, and so did studies in high-income countries, with a modest increase in upper-middle-income countries, particularly China. The entries of lower-middle-income countries (in 2018) and lower-income countries (in 2021) lagged.

### Posting of Study Results on ClinicalTrials.gov

**Figure 6:**
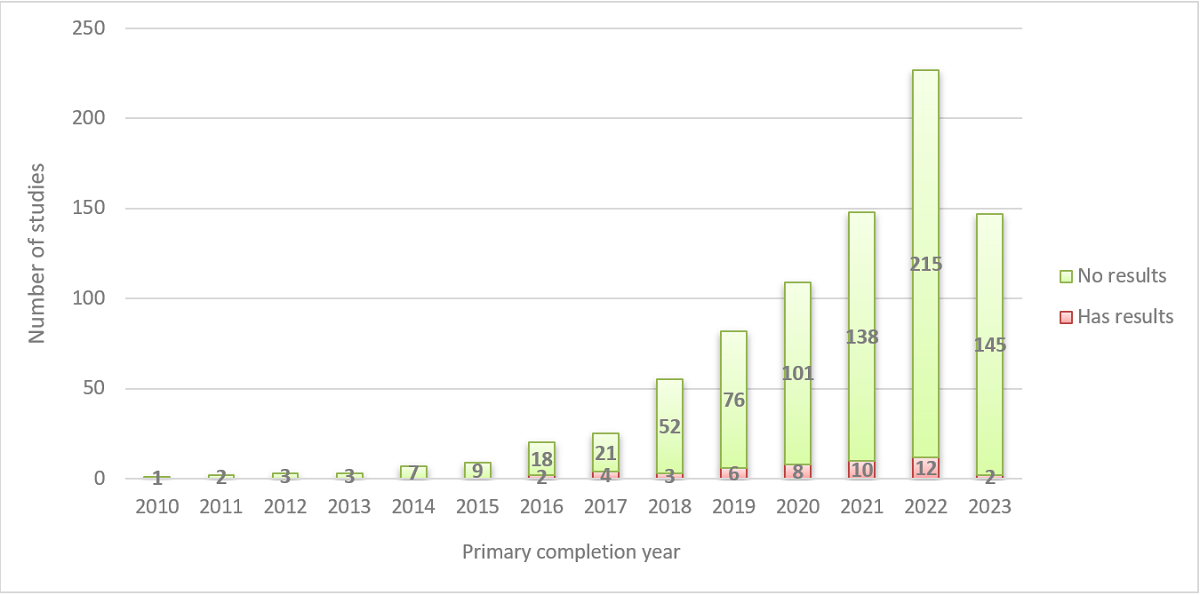
Availability of results posted on ClinicalTrials.gov by primary completion year among completed studies, 2010-2023 (n=838 instead of 842 after excluding 4 studies that reported 2024 as the primary completion year).

Overall, only 5.6% (47/842) of completed studies had results available on ClinicalTrials.gov. Among studies with posted results (n=47), the median time from “primary completion date” to “results first posted date” was 505 days (IQR 399-700; minimum 93, maximum 1200). During 2018-2022, as the rapid increase in AI/ML research became evident, completed studies increased from 55 to 227, but the reporting rate stagnated, remaining between 5.3% and 7.3% (Table 3). Consequently, the number of completed studies without posted results increased (Figure 6).

**Table 3:** Availability of results posted on ClinicalTrials.gov by primary completion year among completed studies, 2010-2023.

Primary completion year	Has results	No results	Total	Percentage of studies with results
2010	0	1	1	0
2011	0	2	2	0
2012	0	3	3	0
2013	0	3	3	0
2014	0	7	7	0
2015	0	9	9	0
2016	2	18	20	10
2017	4	21	25	16
2018	3	52	55	5.5
2019	6	76	82	7.3
2020	8	101	109	7.3
2021	10	138	148	6.8
2022	12	215	227	5.3
2023	2	145	147	1.4
Total	47	791	838^a^	5.6

^a^n=838 instead of 842 after excluding 4 studies that reported 2024 as the primary completion year.

### AI/ML Terms Used in Study Descriptions

The most frequently used term in study descriptions was “artificial intelligence” (37%), followed by “machine learning” (31.4%), “deep learning” (12.3%), “convolutional neural network” (2.6%), and “random forest” (2.1%; Figure S2 in Multimedia Appendix 2). The use of “artificial intelligence,” “machine learning,” and “deep learning” has increased since 2017, especially the former two (Figure S4 in Multimedia Appendix 2).

## Discussion

Of 3106 AI/ML studies over the past 14 years, 63% (n=1951) started in the last 3 years alone; more than half (56.7%; 1762/3106) have set the primary completion date between 2023 and 2050, with a median time of 7 years (IQR 3.5-12) beyond 2023. Thus, our data indicate the general direction of the AI/ML pipeline in the years ahead.

**Principal Findings**

In determining whether the accelerated growth of AI/ML research reflects an increase in desirable study attributes or otherwise, we found uneven growth occurring in three areas: (1) study design, (2) study location, and (3) study results availability. First, the increase in randomized (interventional) and prospective (observational) designs, along with large studies (n>1000), representing a quarter of all studies, may indicate a move toward a prospective comparative evaluation stage. Second, much of the rapid growth in AI/ML studies comes from high-income countries in high-resource settings. Third, the availability of results posted on ClinicalTrials.gov remained limited, and thus, the number of completed studies without posted results increased over time. While the first point seems promising, the other two perpetuate previously raised issues.

### Study Design

Previous research on AI studies registered on ClinicalTrials.gov as of March 2022 concluded that the proportion of trials with prospective (59.92%) and randomized (52.7%) designs was insufficient [1]. We also observed similar proportions (prospective: 58.9%; randomized: 56.2%) as of December 2023. However, from 2017 to 2023, the proportions of RCT and prospective designs increased by 42.1% and 22.6%, respectively, while retrospective and cross-sectional studies decreased by 11.9% and 28.5%, respectively. This is deemed an improvement compared to the period when retrospective assessments were dominant [11].

Although the choice of RCTs depends on the stage of development, the “phase” data, more aligned with drug development, was mostly “not applicable” (93%) in our data (Table 1). Referring to a stage-specific reporting guideline for the early and live clinical evaluation of decision-support systems based on AI, which compares development pathways among drugs, AI, and surgical innovation [25], the recent increase in prospective and randomized designs may be a reflection of enough studies progressing from the in silico evaluation stage to the early live clinical evaluation stage (trial phase 1-2 in drugs) and the comparative prospective evaluation stage (trial phase 3 in drugs).

### Enrollment (Sample Size)

AI models, especially deep learning models, often require large datasets to achieve robust performance [26], with sample sizes above 1000 being common. This was empirically reflected in our data overall. When compared to non-AI/ML trials registered on ClinicalTrials.gov during 2000-2019 [23,24], the proportion of smaller trials (1-100) was lower, and the proportion of larger trials (>1000) was higher in our data (Figure S6 in Multimedia Appendix 2). Furthermore, 16.1% (187/1163) of trials had a sample size of >1000, 72.2% (135/187) of which were not completed yet. Similarly, 19.8% of RCTs (129/653, excluding withdrawn) were in the >1000 category, but 68.2% (88/129) were still ongoing. Thus, the majority of large AI/ML trials in our data are still in the pipeline and yet to be captured in future systematic reviews.

### Lead Sponsor Sector

Our reclassification of the “other” groups revealed the strong presence of hospital/clinic or academic sponsorship over industry (Figure 3). The hospital/clinic or academic sectors might be more likely than the industry to engage in AI/ML research even if it lacks commercialization potential. The higher representation of hospitals/clinics and academia might reflect a high volume of nonproprietary AI/ML.

### The Underrepresented

We examined two areas. First, the underrepresentation of women in research [27,28] is well documented. In this study, we found a gradual increase in women-targeted studies over time. Second, 75.3% (2340/3106) of AI/ML studies were conducted in high-income countries, and only 2.8% (88/3106) and 0.1% (3/3106) were conducted in lower-middle-income and low-income countries, respectively. If AI/ML models poorly generalize to people other than those whose data were used to train the algorithms, it may exacerbate existing disparities. While external validation and model recalibration may help mitigate health care inequities due to data disparity [14], inappropriate external validation (and the lack thereof) in AI/ML research was already highlighted [9,29,30]. Validation using unseen data is particularly important in models built with single-center data [31], and most of our sample (77.2%; 2177/2821) indeed comprised single-center studies. However, external validation was mentioned in only 47 of 3106 studies (1.5%). Of these 47, only 9 (19.1%) were conducted in non–high-income countries: China (n=6), Argentina (n=1), Ecuador (n=1), and Russia (n=1), as part of a 10-country study (all were high-income countries, except Russia).

### Availability of Results on ClinicalTrials.gov

While the number of newly initiated studies increased, the number of completed studies without posted results also increased. The limited reporting of even basic results on ClinicalTrials.gov contrasts with the rapid advancements in the field and the registry’s stated purpose of registration and result submission, which includes mitigating publication bias and outcome reporting bias [32].

Outside the AI/ML field, high rates of results dissemination on ClinicalTrials.gov have been reported, including for cancer trials and small studies [33]. With AI/ML research results more accessible on ClinicalTrials.gov, the registry can play a crucial role in reducing publication bias, making it a valuable resource for future systematic reviews or meta-analyses. Although posting results on ClinicalTrials.gov means disseminating data without independent scientific review, summary results are reported in structured tables, and the results information is devoid of conclusions or “spin,” as the system requires objective data rather than subjective narratives [34]. This allows for broad scrutiny by the scientific community, the public, or competitors. Although results posting on ClinicalTrials.gov is mandated only for certain types of trials, all registered studies are eligible for results submission, and the responsibility of providing knowledge given the use of human data should equally apply to both trials and observational studies.

### Limitations

Our study limitations are as follows. The first is representativeness. Sponsors of FDA-regulated studies and trials with sites in the United States are required to submit information to ClinicalTrials.gov [35]. In our sample, only 7.6% (235/3106) of studies were FDA regulated, while 78.2% (2429/3106) did not involve a US site. When registration is voluntary, it is hard to determine what the data are truly representative of. Interventional trials are more likely to be registered in a public registry due to this being a requirement of the International Committee of Medical Journal Editors as a condition of consideration for publication [36]. However, as this requirement started in 2005 and precedes our data cutoffs (2010-2023), it is unlikely to have influenced our findings (RCTs were infrequent until 2017). Moreover, registered studies would be more likely to share results than unregistered ones, but the reporting rate in our sample was unexpectedly low. Nevertheless, by evaluating trends over time under the same representativeness constraints, we made the best use of the most recent data available.

Second, as we prioritized sensitivity over specificity, our sample may have included studies in which AI/ML played a minor role. Identifying such studies would have required manual free-text data mining with prespecified criteria, which was impractical during testing due to substantial variations in reporting quality, length, and clarity. Poor reporting in AI/ML research has already been highlighted [8,9], and AI-specific reporting guidelines and documentation standards now exist to increase awareness of key dimensions that should be described in study protocols (the SPIRIT-AI Extension, based on SPIRIT [Standard Protocol Items: Recommendations for Interventional Trials]) [37], prediction models (the TRIPOD+AI Statement, based on TRIPOD [Transparent Reporting of a Multivariate Prediction Model for Individual Prognosis or Diagnosis]) [38], or the minimum information about clinical AI modeling (the MI-CLAIM checklist) [39]. The same principle could also apply to study descriptions on ClinicalTrials.gov, and more standardized content within the platform would benefit its audience.

### Conclusions

Much of the rapid growth in AI/ML studies comes from high-income countries in high-resource settings, albeit with a modest increase in upper-middle-income countries (mostly China). Lower-middle or low-income countries remain poorly represented. The increase in randomized or prospective designs, along with large studies (n>1000), which represent a quarter of all studies (mostly ongoing), may indicate that enough studies are progressing from the in silico evaluation stage toward a prospective comparative evaluation stage. However, the ongoing limited availability of basic results posted on ClinicalTrials.gov contrasts with the rapid advancements in this field and the public registry’s role in reducing publication and outcome reporting biases.

## Appendix

Multimedia Appendix 1 Technical document.

Multimedia Appendix 2 Supplementary file (Figures, Tables).

## Abbreviations

AI: artificial intelligence
CTTI AACT: Clinical Trials Transformation Initiative Aggregate Analysis of ClinicalTrials.gov
FDA: Food and Drug Administration
GNI: gross national income
MeSH: Medical Subject Headings
MI-CLAIM: minimum information about clinical AI modeling
ML: machine learning
pSQL: PostgreSQL
RCT: randomized controlled trial
SPIRIT: Standard Protocol Items: Recommendations for Interventional Trials
STROBE: Strengthening the Reporting of Observational Studies in Epidemiology
TRIPOD: Transparent Reporting of a Multivariate Prediction Model for Individual Prognosis or Diagnosis

## Data Availability

The search strategy is provided in Multimedia Appendix 2. The pSQL codes used are provided in Multimedia Appendix 1. The datasets generated during this study are available from the corresponding author on reasonable request.
