# Supplementary material for: Studies of Artificial Intelligence/Machine Learning Registered on ClinicalTrials.gov: Cross-Sectional Study With Temporal Trends, 2010-2023
Source: J Med Internet Res. 2024 Oct 25;26:e57750. doi: 10.2196/57750 (PMC11549584; doi:10.2196/57750)
Supplement: Multimedia Appendix 1 [file jmir_v26i1e57750_app1.docx]

**Technical Document**

Contents

[**Technical Document** 1](#_Toc180504330)

[Retrieving ClinicalTrials.gov Data Set (“Main Set”) 1](#_Toc180504331)

[SQL for the “Main” set of studies and variables in analysis. 1](#_Toc180504332)

[Filtering STUDIES table to retrieve relevant studies. 1](#_Toc180504333)

[Main Set, retrieved and calculated variables. 2](#_Toc180504334)

[MeSH further Processing for *Clinical_Specialty* Analysis 3](#_Toc180504335)

[Deriving *Clinical_Specialty* from MeSH Headings 3](#_Toc180504336)

[Deriving *Clinical_Specialty* from *disease* variable 4](#_Toc180504337)

[Combining the two groups 4](#_Toc180504338)

[Appendix 1. SQL 1. Code to extract Study data from ClinicalTrials.gov 5](#_Toc180504339)

[Appendix 2. SQL 2. Code to extract MeSH Headings from ClinicalTrials.gov 11](#_Toc180504340)

# Retrieving ClinicalTrials.gov Data Set (“Main Set”)

A static copy of the Clinical Trials Transformation Initiative (CTTI) Aggregate Analysis of ClinicalTrials.gov (AACT) database was downloaded on February 6, 2024 (pSQL code provided in Supplementary appendices). Our data source was registered studies that were first posted on ClinicalTrials.gov during January 1, 2010 – December 31, 2023.

## SQL for the “Main” set of studies and variables in analysis.

Postgres SQL code for this section is found in Appendix 1 – SQL 1 of this document.
All of the data related to this query comes from tables within the AACT database.

### Filtering STUDIES table to retrieve relevant studies.

The STUDIES table is the primary driver of the SQL. Studies included were posted between 1-Jan-2010 and 31-Dec-2023 (inclusive), using the *start_date* field.

For each study a text string (called *full_search_string*) was created by combining search terms with OR.

Fields searched:
STUDIES.*official_title*, DETAILED_DESCRIPTIONS.*description*, ALL_INTERVENTIONS.*names*, ALL_KEYWORDS.*names*, BRIEF_SUMMARIES.*description*, DESIGN_OUTCOMES.*measure* and DESIGN_OUTCOMES.*description*.

If any of the following terms were found in *full_search_string*, the study was selected as candidates:

'%artificial_intelligence%', '%artificial_neural_network%', '% ai_based%', '%augmented_intelligence%', '%bayes%_network%', '%classification_tree%', '%convolutional_neural_network%', '%deep_learning%', '%deep_neural_network%', '%deep_reinforcement_learning%', '%generative_adversarial_network%', '%gradient_boosting%', '%k_nearest_neighb%', '%machine_learning%', '%multilayer_perceptron%', '%na_ve_bayes%', '%natural_language_processing%', '%random_forest%', '%recurrent_neural_network%', '%regression_tree%', '%reinforcement_learning%', '%supervised_learning%', '%support_vector_machine%', '%swarm_intelligence%', '%unsupervised_learning%', '%xgboost%', '%elastic_net%'

### Main Set, retrieved and calculated variables.

The SQL produced the “Main Set”, a results set that contained 70 variables for each study. The *nct_id* column is the unique identifier for each study in ClinicalTrials.gov. The SQL derived the following variables for each study:

#### funding_source

As the variable *funding* under the ANALYZED_STUDIES table was mostly missing, making it unfit for analysis, we calculated as per the definition provided in the AACT Data Dictionary: “Funding Source Calculated”^[[1]](#footnote-1)^ (as at Oct-23). which used data from the SPONSORS.*lead_or_collaborator* and SPONSORS.*agency_class* columns.

*funding_source* =

If Sponsor is from NIH, or at least one collaborator is from NIH with no Industry sponsor then
funding=NIH.

Otherwise, if Sponsor is from Industry or at least one collaborator is from Industry then
funding=Industry.

Studies with no Industry or NIH Sponsor or collaborators are assigned then
funding=Other.

See Appendix 1 - SQL 1, subquery sub_sponsors for detailed calculations.

#### lead_sponsor and lead_sponsor_agency_class

For each *nct_id*, sponsor record was selected from the SPONSORS table, where the field *lead_or_collaborator* = ‘lead’ was selected (every *nct_id* had one-and-only-one ‘lead’ entry). For this record:

*lead_sponsor* = SPONSORS.*name*

*lead_sponsor_agency_class* = SPONSORS.*agency_class*

See Appendix 1 - SQL 1, subquery sub_sponsors for detailed calculations.

#### results_posted_within_1y

According to the records in ClinicalTrials.gov STUDIES table, were the results posted within 1 year of the study’s completion date.

If STUDIES.*primary_completion_date* + 356) <= STUDIES.*results_first_posted_date* then 1, else 0

# MeSH further Processing for *Clinical_Specialty* Analysis

Studies were grouped into clinical specialties using the Medical Subject Headings (MeSH) largest headings, and where MeSH headings were missing, submitted disease condition terms^[[2]](#footnote-2)^.

## Deriving *Clinical_Specialty* from MeSH Headings

A secondary data extract was run on AACT to obtain the list of *mesh_headings* and the number of MeSH terms under each heading. (see Appendix 2 for the pSQL code)

ClinicalTrials.gov stores the hierarchal MeSH structure in the MESH_TERMS and MESH_HEADINGS tables. Both tables have a *qualifier* column, a three-character value that is used to register the *mesh_heading*. The table BROWSE_CONDITIONS relates each *nct_id* to a series of MeSH terms, but the MESH_HEADING.*qualifier* field is not recorded. Therefore, we created a virtual reference table of MeSH headings and the corresponding *qualifier*. Using this, we could link each MESH_TERMS.*downcase_mesh_term* to its respective *mesh_heading*.

STUDIES
*NCT_ID*

BROWSE_CONDITIONS
*NCT_ID
downcase_mesh_term*

MESH_TERMS
*downcase_mesh_term
qualifier*

MESH_HEADINGS (distinct)
*qualifier
heading*

**Figure 1.** Simplified entity relationships from STUDIES to MESH_HEADINGS tables in AACT database.

Finally, to create the *Clinical_Specialty* variable, for each NCT_ID, we selected only the headings with the maximum number of mesh terms counted (n=2892). Note that some studies appeared multiple times (n=278).

## Deriving *Clinical_Specialty* from *disease* variable

As noted in Tasneem^[[3]](#footnote-3)^, some studies in the AACT database are not allocated MeSH terms (n=570 in the “Main Set”). In these studies, the *disease* variable was used to derive the *Clinical_Specialty* grouping.

## Combining the two groups

The two sets of data relating studies to *Clinical_Specialty* were combined in the Excel workbook.

# Appendix 1. SQL 1. Code to extract Study data from ClinicalTrials.gov

Note: This is an annotated text copy from the actual SQL file called
“**Appendix 1 - SQL 1.pgsql**”.
Please run from the accompanying file instead of copying this text.
Output from this SQL was saved in the spreadsheet tab “**Main Set**”

Select

main.nct_id

, case when main.overall_status = 'Completed' then 'Completed'

when main.overall_status in ('Terminated', 'Withdrawn', 'Suspended')

then 'Stopped'

when main.overall_status in ( 'Recruiting', 'Not yet recruiting'

, 'Active, not recruiting'

, 'Enrolling by invitation')

then 'Active'

else main.overall_status -- eg. 'Unknown Status' or null or new status

end as overall_status_grp

, main.overall_status

-- Important Dates

, extract('Year' from start_date) as start_yr

, main.start_date

, main.primary_completion_date

, coalesce(main.completion_date, main.primary_completion_date)

as completion_Date

, (main.primary_completion_date - main.start_date) as time2primaryComp

, (main.completion_date - main.start_date) as days2comp

, (main.completion_date - main.start_date)/30.44 as months2comp

-- Results Published. (Only CTG perspective)

, main.Results_First_Posted_date

, (main.completion_date + 365) as Basic_results_within_1y_after_Comp_Date

, (case calculated_values.were_results_reported WHEN false then 'No results'

else 'Has results' end) as Results

, calculated_values.months_to_report_results

, (case when (main.completion_date+365) <= main.Results_First_Posted_date

then 1 else 0 end) as Result_posted_within_1y

, main.study_First_Posted_date

, main.Last_Update_Posted_date

-- References/Citations

, (select count(*) from study_references as X

where x.nct_id = main.nct_id

and reference_type in ('derived', 'result')

) as nr_ctg_publications

, main.official_title

, main.brief_title

, main.phase

, main.enrollment

, case when enrollment is NULL then 'Error'

when enrollment <= 0 then 'Error'

when enrollment <= 100 then '① <=100'

when enrollment <= 1000 then '② 101-1,000'

when enrollment <= 5000 then '③ 1,001-5,000'

when enrollment <= 10000 then '④ 5,001-10,000'

when enrollment <= 20000 then '⑤ 10,001-20,000'

when enrollment <= 30000 then '⑥ 20,001-30,000'

else '⑦ >30,000'

end as enroll_class

, coalesce(main.number_of_arms, 0) as nr_arms

, main.study_type

, main.interventions_list

, main.brief_summary

, (select names from all_conditions as X

where x.nct_id = main.nct_id) as disease

, (select names from all_design_outcomes as X

where x.nct_id = main.nct_id) as Outcome_measures

, (select names from all_primary_outcome_measures as X

where x.nct_id = main.nct_id) as primary_outcome_measures

, main.Primary_Outcomes_Details

, (select names from all_secondary_outcome_measures as X

where x.nct_id = main.nct_id) as Secondary_outcome_measures

, (select names from all_group_types as X

where x.nct_id = main.nct_id) as group_types

-- Variables related to location, facilities, countries

, coalesce(calculated_values.number_of_facilities, 0) as nr_facilites

, (select names from all_facilities as X

where x.nct_id = main.nct_id) as facilities_list

, (select names from all_countries as X

where x.nct_id = main.nct_id) as countries_list

, 'Replace with xlFormula' as Region

, sub_sponsors.lead_sponsor

, sub_sponsors.lead_sponsor_agency_class

, sub_sponsors.funding_source

, (select string_agg(distinct non_inferiority_type, '|')

from outcome_analyses as X

where x.nct_id = main.nct_id) as non_inferiority_type_list

, eligibilities.gender

, eligibilities.minimum_age as Minimum_age

, eligibilities.maximum_age as maximum_age

, eligibilities.healthy_volunteers

, eligibilities.population as study_pop

-- From Designs table

, designs.allocation

, designs.intervention_model

, designs.primary_purpose

, designs.time_perspective

, designs.masking

, (case designs.allocation WHEN 'Randomized' then 1 else 0 end) as Randomized

, main.is_fda_regulated_drug as fda_drug

, main.is_fda_regulated_device as fda_device

, main.plan_to_share_ipd

--- FILTER SELECTION FLAGS --- -----------------------------------------

-- Identify which filter was hit to include this row.

-- Doesn't show which text field in the key was hit though.

, case when main.full_search_string like '%artificial_intelligence%' then 1 else 0 end as Artificial_intelligence

, case when main.full_search_string like '%artificial_neural_network%' then 1 else 0 end as artificial_neural_network

, case when main.full_search_string like '% ai_based%' then 1 else 0 end as AI_based

, case when main.full_search_string like '%augmented_intelligence%' then 1 else 0 end as augmented_intelligence

, case when main.full_search_string like '%bayes%_network%' then 1 else 0 end as bayes_network

, case when main.full_search_string like '%classification_tree%' then 1 else 0 end as classification_tree

, case when main.full_search_string like '%convolutional_neural_network%' then 1 else 0 end as convolutional_neural_network

, case when main.full_search_string like '%deep_learning%' then 1 else 0 end as deep_learning

, case when main.full_search_string like '%deep_neural_network%' then 1 else 0 end as deep_neural_network

, case when main.full_search_string like '%deep_reinforcement_learning%' then 1 else 0 end as deep_reinforcement_learning

, case when main.full_search_string like '%generative_adversarial_network%' then 1 else 0 end as generative_adversarial_network

, case when main.full_search_string like '%gradient_boosting%' then 1 else 0 end as gradient_boosting

, case when main.full_search_string like '%k_nearest_neighb%' then 1 else 0 end as k_nearest_neighb

, case when main.full_search_string like '%machine_learning%' then 1 else 0 end as machine_learning

, case when main.full_search_string like '%multilayer_perceptron%' then 1 else 0 end as multilayer_perceptron

, case when main.full_search_string like '%na_ve_bayes%' then 1 else 0 end as na_ve_bayes

, case when main.full_search_string like '%natural_language_processing%' then 1 else 0 end as natural_language_processing

, case when main.full_search_string like '%random_forest%' then 1 else 0 end as random_forest

, case when main.full_search_string like '%recurrent_neural_network%' then 1 else 0 end as recurrent_neural_network

, case when main.full_search_string like '%regression_tree%' then 1 else 0 end as regression_tree

, case when main.full_search_string like '%reinforcement_learning%' then 1 else 0 end as reinforcement_learning

, case when main.full_search_string like '%supervised_learning%' then 1 else 0 end as supervised_learning

, case when main.full_search_string like '%support_vector_machine%' then 1 else 0 end as support_vector_machine

, case when main.full_search_string like '%swarm_intelligence%' then 1 else 0 end as swarm_intelligence

, case when main.full_search_string like '%unsupervised_learning%' then 1 else 0 end as unsupervised_learning

, case when main.full_search_string like '%xgboost%' then 1 else 0 end as xgboost

, case when main.full_search_string like '%elastic_net%' then 1 else 0 end as elastic_net

, main.keywords_by_spo

, main.Mesh_terms_conditions_list

-- Full matrix of MeSH headings used for each study

, mesh_list.*

-- MeSH heading most often found in this study

, mesh_main_contrib.mesh_largest_qualifier

, mesh_main_contrib.mesh_largest_heading

-- Type of Study Flags -- Helps identify irrelevant studies.

, (case when main.full_search_string like '% future %' then 1 end) as f_future

, (case when main.full_search_string like '% previous% %'

or main.full_search_string like '% past %' then 1 end) as f_prev_past

, (case when main.full_search_string like '% protocol% %' then 1 end) as f_protocol

, (case when main.full_search_string like '% design% %' then 1 end) as f_design

, (case when main.full_search_string like '% review% %' then 1 end) as f_review

, (case when main.full_search_string like '% systematic_review% %' then 1 end) as f_sys_review

, (case when main.full_search_string like '% scoping_review% %' then 1 end) as f_scop_review

, (case when main.full_search_string like '% literature review% %' then 1 end) as f_lit_review

, (case when main.full_search_string like '% meta_analys% %' then 1 end) as f_meta_analysis

, (case when main.full_search_string like '% propensity_score% %' then 1 end) as f_propensity_score

-- , main.full_search_string

from (

-- Main Query Driver. ***********************************************

-- a) Uses STUDIES table

-- b) Builds a search-key string from several fields and other tables.

-- c) full_search_string is used in both the "select"

-- and "where" parts of the overall query.

select regexp_replace(

regexp_replace(

lower(

coalesce(studies.official_title, 'Missing_OT')

|| '~' || coalesce(detailed_descriptions.description, 'Missing_DD')

|| '~' || coalesce(all_interventions.names, 'Missing_I')

|| '~' || coalesce(all_keywords.names, 'Missing_Kw')

|| '~' || coalesce(all_browse_conditions.names, 'Missing_BC')

|| '~' || coalesce(brief_summaries.description, 'Missing_BS')

|| '~' || coalesce(all_outcomes.Primary_Outcomes_Details, 'Missing_AO')

), '[^\w\s]', '', 'g'

), '\s+', ' ', 'g'

) as full_search_string

, studies.*

, all_interventions.names as interventions_list

, all_keywords.names as keywords_by_spo

, all_browse_conditions.names as Mesh_terms_conditions_list

, brief_summaries.description as brief_summary

, all_outcomes.Primary_Outcomes_Details

from studies

LEFT JOIN detailed_descriptions

on studies.nct_id = detailed_descriptions.nct_id

LEFT JOIN all_interventions

on studies.nct_id = all_interventions.nct_id

LEFT JOIN all_keywords

on studies.nct_id = all_keywords.nct_id

LEFT JOIN all_browse_conditions

on studies.nct_id = all_browse_conditions.nct_id

left join brief_summaries

on studies.nct_id = brief_summaries.nct_id

left join (

select nct_id, string_agg(measure, '~')

|| '~' || string_agg(description, '~') as Primary_Outcomes_Details

from design_outcomes

where outcome_type = 'primary'

group by nct_id

) as all_outcomes

on studies.nct_id = all_outcomes.nct_id

) as MAIN

-- End Main Query Driver. **************************************************

LEFT JOIN designs on main.nct_id = designs.nct_id

LEFT JOIN calculated_values on main.nct_id = calculated_values.nct_id

LEFT JOIN eligibilities on main.nct_id = eligibilities.nct_id

-- 2023-10-01 The subquery below replicates the rule in AACT data dictionary.

-- Needed to replicate because data in table

-- proj_results_reporting.analyzed_studies is too old and invalid

-- Source of rule:

-- https://aact.ctti-clinicaltrials.org/data_dictionary

-- (search for column="funding")

-- Rule Text (as at Oct-23):

-- Funding Source=

-- Derived from Sponsor and Collaborator information.

-- * If Sponsor is from NIH, or at least one collaborator is from NIH with no Industry sponsor then funding=NIH.

-- * Otherwise if Sponsor is from Industry or at least one collaborator is from Industry then funding=Industry.

-- * Studies with no Industry or NIH Sponsor or collaborators are assigned funding=Other.

LEFT JOIN (

select nct_id

, case when ruleLead > 0 then lead_sponsor_agency_class

when ruleNih > 0 then 'NIH'

when ruleIndustry > 0 then 'INDUSTRY'

else 'Other'

end as funding_Source

, lead_sponsor

, lead_sponsor_agency_class

, nr_sponsors

from (

select nct_id, count(*) nr_Sponsors

, count(case when lead_or_collaborator = 'lead'

and agency_class in ('NIH', 'INDUSTRY') then 1 end) as ruleLead

, count(case when lead_or_collaborator = 'collaborator'

and agency_class = 'NIH' then 1 end) as ruleNih

, count(case when lead_or_collaborator = 'collaborator'

and agency_class = 'INDUSTRY' then 1 end) as ruleIndustry

, max(case when lead_or_collaborator = 'lead' then name end) as lead_sponsor

, max(case when lead_or_collaborator = 'lead' then agency_class end) as lead_sponsor_agency_class

from sponsors

group by nct_id

) as sub_Inner

) as sub_sponsors on main.nct_id = sub_sponsors.nct_id

LEFT JOIN (

select bc.nct_id

, string_agg(distinct mh.heading, '|') as mesh_Heading_list

-- !!! Note the order of this will be alphabetical and NOT match the order of mesh_Heading_list

, string_agg(distinct mh.qualifier, '|') as mesh_qualifier_list

, count(case when mt.qualifier = 'G17' then 1 end) as G17

, count(case when mt.qualifier = 'L01' then 1 end) as L01

from browse_conditions as BC

join mesh_terms as MT

on bc.downcase_mesh_term = mt.downcase_mesh_term

left join (select qualifier, min(heading) heading

from mesh_headings

where length(qualifier) = 3

group by qualifier

) as MH on mt.qualifier = mh.qualifier

group by bc.nct_id

) as mesh_list on main.nct_id = mesh_list.nct_id

-- Work out what the main MeSH Heading contributor is.

-- Ended up not using this field. See SQL2 instead.

LEFT JOIN (

select distinct on (bc.nct_id) nct_id

, mt.qualifier as MeSH_largest_qualifier

, max(mh.heading) as MeSH_largest_heading

, count(*) as nr_terms

from browse_conditions as BC

join mesh_terms as MT

on mt.downcase_mesh_term = bc.downcase_mesh_term

join (select distinct qualifier, heading

from mesh_headings

) as MH on mt.qualifier = mh.qualifier

group by bc.nct_id, mt.qualifier

order by bc.nct_id, nr_terms desc

) as mesh_main_contrib on main.nct_id = mesh_main_contrib.nct_id

-- ===========================================================================

where

main.start_date >= '2010-01-01'

and main.start_date < '2024-01-01'

-- Main filter for ai/ml keywords.

and (

main.full_search_string like '%artificial_intelligence%'

or main.full_search_string like '%artificial_neural_network%'

or main.full_search_string like '% ai_based%'

or main.full_search_string like '%augmented_intelligence%'

or main.full_search_string like '%bayes%_network%'

or main.full_search_string like '%classification_tree%'

or main.full_search_string like '%convolutional_neural_network%'

or main.full_search_string like '%deep_learning%'

or main.full_search_string like '%deep_neural_network%'

or main.full_search_string like '%deep_reinforcement_learning%'

or main.full_search_string like '%generative_adversarial_network%'

or main.full_search_string like '%gradient_boosting%'

or main.full_search_string like '%k_nearest_neighb%'

or main.full_search_string like '%machine_learning%'

or main.full_search_string like '%multilayer_perceptron%'

or main.full_search_string like '%na_ve_bayes%'

or main.full_search_string like '%natural_language_processing%'

or main.full_search_string like '%random_forest%'

or main.full_search_string like '%recurrent_neural_network%'

or main.full_search_string like '%regression_tree%'

or main.full_search_string like '%reinforcement_learning%'

or main.full_search_string like '%supervised_learning%'

or main.full_search_string like '%support_vector_machine%'

or main.full_search_string like '%swarm_intelligence%'

or main.full_search_string like '%unsupervised_learning%'

or main.full_search_string like '%xgboost%'

or main.full_search_string like '%elastic_net%' 02-06

)

ORDER BY main.nct_id DESC

# Appendix 2. SQL 2. Code to extract MeSH Headings from ClinicalTrials.gov

This is an annotated text copy from the actual SQL file called
“**Appendix 2 - SQL 2.pgsql**”.
Please run from the accompanying file instead of copying this text.
Output from this SQL was saved in the spreadsheet tab “**Clinical_Specialty.xlsx**”

create temporary table set1 as (

select nct_id, start_yr, qualifier, heading, count(*) nr_terms

from (

select s.nct_id

, extract('Year' from s.start_date) as start_yr

, bc.downcase_mesh_term, mesh_type

, mh.qualifier, mh.heading

from studies S

join browse_conditions BC on bc.nct_id = s.nct_id

join mesh_terms MT on mt.downcase_mesh_term = bc.downcase_mesh_term

join (select distinct qualifier, heading

from mesh_headings

) as MH on mt.qualifier = mh.qualifier

where s.nct_id in ('x'

-- List of all NCT_IDs from Main Set

,'NCT01066208', 'NCT01177774', 'NCT01246882', 'NCT01286636', 'NCT01302626', 'NCT01338116', 'NCT01398072', 'NCT01442896', 'NCT01448694', 'NCT01505478', 'NCT01577394', 'NCT01586520'

… etc ( see actual SQL file for the list.

)

) sub

group by nct_id, start_yr, qualifier, heading

ORDER BY nct_id DESC

);

-- ============================================================================

select *

from set1

where (nct_id, nr_terms) in (

select nct_id, max(nr_terms)

from set1

group by nct_id

)

;

1. See definition at https://aact.ctti-clinicaltrials.org/data_dictionary (search for column="funding") [↑](#footnote-ref-1)
2. Califf RM, Zarin DA, Kramer JM, Sherman RE, Aberle LH, Tasneem A. Characteristics of clinical trials registered in ClinicalTrials.gov, 2007-2010. Jama. 2012;307(17):1838-47. doi: 10.1001/jama.2012.3424. PubMed PMID: 22550198. [↑](#footnote-ref-2)
3. Tasneem A, Aberle L, Ananth H, Chakraborty S, Chiswell K, et al. (2012) The Database for Aggregate Analysis of ClinicalTrials.gov (AACT) and Subsequent Regrouping by Clinical Specialty. PLoS ONE 7(3): e33677. doi:10.1371/journal.pone.0033677 [↑](#footnote-ref-3)
