# Supplementary material for: Studies of Artificial Intelligence/Machine Learning Registered on ClinicalTrials.gov: Cross-Sectional Study With Temporal Trends, 2010-2023
Source: J Med Internet Res. 2024 Oct 25;26:e57750. doi: 10.2196/57750 (PMC11549584; doi:10.2196/57750)
Supplement: Multimedia Appendix 2 [file jmir_v26i1e57750_app2.docx]

**Table S1.** Search strategy.

| Date searched | 6 February 2024 |
| --- | --- |
| Source data | Clinical Trials Transformation Initiative \|  Aggregate Analysis of ClinicalTrials.gov (AACT) database |
| Table name | STUDIES to retrieve relevant studies |
| Table columns searched | official title, brief title, brief summary, interventions, detailed descriptions, primary outcome, or keywords |
| Period | Start date: from 1-Jan-2010 to 31-Dec-2023 |
| Searched data fields | STUDIES.official_title, DETAILED_DESCRIPTIONS.description, ALL_INTERVENTIONS.names, ALL_KEYWORDS.names, ALL_BROWSE_CONDITIONS.names, BRIEF_SUMMARIES.description, DESIGN_OUTCOMES.measure and DESIGN_OUTCOMES.description (where ‘design_outcome”=primary outcome) |

| Text search - combined with OR |
| --- |
| ai-based  artificial intelligence  artificial neural network*  augmented intelligence  bayes* network*  classification tree*  convolutional neural network*  deep learning  deep neural network*  deep reinforcement learning  elastic net  generative adversarial network*  gradient boosting  k nearest neighb*  machine learning  multilayer perceptron*  naïve bayes or naive bayes  natural language processing*  random forest  recurrent neural network*  regression tree  reinforcement learning  supervised learning  support vector machine*  unsupervised learning  XGBoost |

The asterisk (*) denotes a wildcard character.

Entire SQL codes are provided in the **Online Supplementary File 2**.

**Table S2.** Study Location – Country, 2010-2023.

| **Rank** | **Country** | **Single-country studies,**  **n** | **Single & multi-country studies,**  **n** | | **Income group (GNI per capita)** |
| --- | --- | --- | --- | --- | --- |
| 1 | United States | 633 | 677 | 21.8% | High income |
| 2 | China | 541 | 550 | 17.7% | Upper middle income |
| 3 | France | 185 | 205 | 6.6% | High income |
| 4 | United Kingdom | 170 | 193 | 6.2% | High income |
| 5 | Italy | 124 | 162 | 5.2% | High income |
| 6 | Taiwan | 107 | 111 | 3.6% | High income |
| 7 | Canada | 98 | 122 | 3.9% | High income |
| 8 | Spain | 92 | 125 | 4.0% | High income |
| 9 | Korea | 72 | 76 | 2.4% | High income |
| 10 | Germany | 63 | 89 | 2.9% | High income |
| 11 | Netherlands | 53 | 71 | 2.3% | High income |
| 12 | Switzerland | 52 | 60 | 1.9% | High income |
| 13 | Turkey | 45 | 50 | 1.6% | Upper middle income |
| 14 | Belgium | 42 | 52 | 1.7% | High income |
| 15 | Hong Kong | 38 | 40 | 1.3% | High income |
| 16 | Sweden | 37 | 50 | 1.6% | High income |
| 17 | Israel | 34 | 45 | 1.4% | High income |
| 18 | Singapore | 33 | 36 | 1.2% | High income |
| 19 | Denmark | 32 | 38 | 1.2% | High income |
| 20 | Austria | 21 | 29 | 0.9% | High income |
| 21 | Norway | 21 | 25 | 0.8% | High income |
| 22 | Egypt | 20 | 24 | 0.8% | Lower middle income |
| 23 | India | 17 | 23 | 0.7% | Lower middle income |
| 24 | Greece | 15 | 20 | 0.6% | High income |
| 25 | Brazil | 13 | 16 | 0.5% | Upper middle income |
| 26 | Poland | 11 | 18 | 0.6% | High income |
| 27 | Russia | 11 | 14 | 0.5% | Upper middle income |
| 28 | Finland | 9 | 12 | 0.4% | High income |
| 29 | Australia | 8 | 15 | 0.5% | High income |
| 30 | Pakistan | 8 | 8 | 0.3% | Lower middle income |
| 31 | Slovenia | 6 | 8 | 0.3% | High income |
| 32 | Ecuador | 5 | 6 | 0.2% | Upper middle income |
| 33 | Mexico | 5 | 11 | 0.4% | Upper middle income |
| 34 | Portugal | 5 | 12 | 0.4% | High income |
| 35 | Romania | 5 | 8 | 0.3% | High income |
| 36 | Bangladesh | 4 | 4 | 0.1% | Lower middle income |
| 37 | Czechia | 4 | 7 | 0.2% | High income |
| 38 | Hungary | 4 | 5 | 0.2% | High income |
| 39 | Indonesia | 4 | 4 | 0.1% | Upper middle income |
| 40 | Ireland | 4 | 8 | 0.3% | High income |
| 41 | Thailand | 4 | 5 | 0.2% | Upper middle income |
| 42 | Argentina | 3 | 5 | 0.2% | Upper middle income |
| 43 | Tanzania | 3 | 4 | 0.1% | Lower middle income |
| 44 | Chile | 2 | 2 | 0.1% | High income |
| 45 | Iran | 2 | 2 | 0.1% | Lower middle income |
| 46 | Japan | 2 | 4 | 0.1% | High income |
| 47 | Kenya | 2 | 4 | 0.1% | Lower middle income |
| 48 | Malaysia | 2 | 4 | 0.1% | Upper middle income |
| 49 | Nigeria | 2 | 3 | 0.1% | Lower middle income |
| 50 | South Africa | 2 | 6 | 0.2% | Upper middle income |
| 51 | Belarus | 1 | 1 | 0.0% | Upper middle income |
| 52 | Bolivia | 1 | 1 | 0.0% | Lower middle income |
| 53 | Burkina Faso | 1 | 1 | 0.0% | Low income |
| 54 | Cameroon | 1 | 1 | 0.0% | Lower middle income |
| 55 | Colombia | 1 | 2 | 0.1% | Upper middle income |
| 56 | Croatia | 1 | 2 | 0.1% | High income |
| 57 | Ethiopia | 1 | 1 | 0.0% | Low income |
| 58 | Lebanon | 1 | 1 | 0.0% | Lower middle income |
| 59 | Lithuania | 1 | 4 | 0.1% | High income |
| 60 | Luxembourg | 1 | 1 | 0.0% | High income |
| 61 | Malta | 1 | 2 | 0.1% | High income |
| 62 | Morocco | 1 | 1 | 0.0% | Lower middle income |
| 63 | Paraguay | 1 | 1 | 0.0% | Upper middle income |
| 64 | Qatar | 1 | 1 | 0.0% | High income |
| 65 | Rwanda | 1 | 1 | 0.0% | Low income |
| 66 | Slovakia | 1 | 2 | 0.1% | High income |
| 67 | Ukraine | 1 | 1 | 0.0% | Lower middle income |
| 68 | United Arab Emirates | 1 | 3 | 0.1% | High income |
| 69 | Vietnam | 1 | 5 | 0.2% | Lower middle income |
| 70 | Zambia | 1 | 3 | 0.1% | Lower middle income |
| 71 | Zimbabwe | 1 | 3 | 0.1% | Lower middle income |
|  | Total | 2696 | 3106 | 100% |  |

GNI: gross national income. National income group classification as per the World Bank.

Table S3. Clinical Specialties by start year, 2010-2023.

|  | **2010** | **2011** | **2012** | **2013** | **2014** | **2015** | **2016** | **2017** | **2018** | **2019** | **2020** | **2021** | **2022** | **2023** | **Total** |
| --- | --- | --- | --- | --- | --- | --- | --- | --- | --- | --- | --- | --- | --- | --- | --- |
| Neoplasms | 4 | 1 | 1 | 1 | 5 | 7 | 5 | 14 | 32 | 40 | 49 | 89 | 92 | 80 | 420 |
| Nervous System Diseases | 1 | 4 | 3 | 6 | 1 | 6 | 9 | 13 | 22 | 46 | 57 | 78 | 66 | 83 | 395 |
| Cardiovascular Diseases |  | 3 | 1 | 1 | 1 | 6 | 9 | 9 | 18 | 29 | 49 | 75 | 65 | 90 | 356 |
| Pathological Conditions |  | 2 | 3 | 4 | 3 | 3 | 5 | 9 | 18 | 22 | 42 | 61 | 84 | 69 | 325 |
| Respiratory Tract Diseases | 2 | 2 | 2 | 2 | 1 | 3 | 1 | 2 | 16 | 14 | 72 | 50 | 52 | 56 | 275 |
| Digestive System Diseases | 2 | 1 | 1 | 1 |  |  | 3 | 5 | 10 | 22 | 38 | 48 | 60 | 62 | 253 |
| Mental Disorders |  |  |  | 1 | 2 | 7 | 13 | 6 | 16 | 19 | 26 | 28 | 52 | 49 | 219 |
| Endocrine, nutritional, or metabolic | 2 |  |  | 1 | 1 | 2 | 6 | 5 | 16 | 11 | 18 | 29 | 39 | 31 | 161 |
| Female Urogenital Diseases and Pregnancy Complications |  | 1 |  |  | 1 | 1 | 1 | 1 | 6 | 9 | 13 | 31 | 41 | 33 | 138 |
| Skin and Connective Tissue Diseases |  |  |  |  | 1 | 3 | 2 | 2 | 8 | 4 | 14 | 19 | 21 | 15 | 89 |
| Eye Diseases |  | 2 | 2 | 3 | 2 | 1 | 1 | 4 | 6 | 6 | 14 | 14 | 11 | 13 | 79 |
| Musculoskeletal Diseases |  |  | 3 |  |  | 1 |  | 2 | 5 | 2 | 5 | 16 | 10 | 16 | 60 |
| Behavior and Behavior Mechanisms |  | 1 |  |  | 2 | 3 | 2 | 3 | 6 | 4 | 3 | 10 | 10 | 12 | 56 |
| Male Urogenital Diseases |  | 1 |  |  |  | 1 |  | 1 | 4 | 2 | 6 | 7 | 13 | 13 | 48 |
| Surgical Procedures, Operative |  |  | 1 | 1 |  |  | 1 | 1 |  | 5 | 4 | 6 | 7 | 12 | 38 |
| Wounds and Injuries |  |  |  |  |  |  |  | 1 | 3 | 1 | 8 | 8 | 9 | 4 | 34 |
| Chemically-Induced Disorders |  |  |  |  |  | 3 | 2 | 3 |  | 2 | 7 | 5 | 6 | 6 | 34 |
| Bacterial Infections and Mycoses |  |  |  |  |  |  | 1 |  | 1 | 1 | 6 | 11 | 5 | 8 | 33 |
| Hemic and Lymphatic Diseases |  |  |  |  | 1 |  | 1 |  | 4 | 3 | 3 | 8 | 8 | 3 | 31 |
| Stomatognathic Diseases |  |  |  |  |  |  |  |  | 2 | 2 | 3 | 5 | 8 | 8 | 28 |
| Radiology (non-organ specific) |  |  |  |  |  |  | 1 |  | 2 | 4 | 7 | 5 | 4 | 3 | 26 |
| Otorhinolaryngologic Diseases |  |  |  |  |  |  | 1 |  |  | 1 | 1 | 7 | 8 | 4 | 22 |
| Physiological Phenomena |  |  |  |  |  |  | 1 |  | 2 | 3 | 2 | 2 | 7 | 4 | 21 |
| Immune System Diseases |  |  |  |  |  |  | 1 |  | 1 | 6 | 3 | 1 | 1 | 6 | 19 |
| Congenital, Hereditary, and Neonatal Diseases |  |  |  | 1 |  |  |  |  |  | 2 | 2 | 1 | 9 | 3 | 18 |
| Virus Diseases | 1 |  |  |  |  |  |  |  |  | 1 | 6 | 2 | 1 | 6 | 17 |
| Critical Care |  |  | 1 |  |  |  |  | 1 |  | 2 | 2 |  | 3 | 5 | 14 |
| Urology/Nephrology |  |  | 1 |  |  |  | 1 |  | 1 |  |  |  | 4 | 6 | 13 |
| Geriatrics |  |  |  |  |  |  | 1 | 1 | 3 |  |  | 2 | 1 | 3 | 11 |
| Environment and Public Health |  |  | 1 |  |  |  |  |  |  |  |  |  | 4 | 2 | 7 |
| Circulatory and Respiratory Physiological Phenomena |  |  |  |  |  |  |  |  |  |  | 2 | 1 | 2 |  | 5 |

**Table S4.** Sample size by lead sponsor sector, 2010-2023.

| Interventional Studies (n=1163)† | | | | | | | | | | | | | | | | |
| --- | --- | --- | --- | --- | --- | --- | --- | --- | --- | --- | --- | --- | --- | --- | --- | --- |
|  | **Hospital/Clinic** | | **Academia** | | **Industry** | | **NPO** | | **Individual** | | **Government** | | **Unknown** | | **Total** | |
|  | **n** | **%** | **n** | **%** | **n** | **%** | **n** | **%** | **n** | **%** | **n** | **%** | **n** | **%** | **n** | **%** |
| 1-100 | 189 | 42.2 | 167 | 43.6 | 66 | 44.9 | 41 | 43.6 | 25 | 42.4 | 9 | 33.3 |  | 0.0 | 497 | 42.7 |
| 101-1,000 | 190 | 42.4 | 163 | 42.6 | 57 | 38.8 | 35 | 37.2 | 23 | 39.0 | 8 | 29.6 | 3 | 60.0 | 479 | 41.2 |
| 1,001-5,000 | 51 | 11.4 | 32 | 8.4 | 15 | 10.2 | 9 | 9.6 | 7 | 11.9 | 2 | 7.4 | 2 | 40.0 | 118 | 10.1 |
| 5,001-10,000 | 10 | 2.2 | 6 | 1.6 | 2 | 1.4 | 3 | 3.2 | 3 | 5.1 | 1 | 3.7 | 0 | 0.0 | 25 | 2.1 |
| 10,001-20,000 | 2 | 0.4 | 2 | 0.5 | 0 | 0.0 | 1 | 1.1 | 1 | 1.7 | 2 | 7.4 | 0 | 0.0 | 8 | 0.7 |
| 20,001-30,000 | 1 | 0.2 | 3 | 0.8 | 1 | 0.7 | 1 | 1.1 | 0 | 0.0 | 1 | 3.7 | 0 | 0.0 | 7 | 0.6 |
| >30,000 | 5 | 1.1 | 10 | 2.6 | 6 | 4.1 | 4 | 4.3 | 0 | 0.0 | 4 | 14.8 | 0 | 0.0 | 29 | 2.5 |
| Total | 448 | 100 | 383 | 100 | 147 | 100 | 94 | 100 | 59 | 100 | 27 | 100 | 5 | 100 | 1163 | 100 |
| Observational Studies (n=1898)† | | | | | | | | | | | | | | | | |
|  | **Hospital/Clinic** | | **Academia** | | **Industry** | | **NPO** | | **Individual** | | **Government** | | **Unknown** | | **Total** | |
|  | **n** | **%** | **n** | **%** | **n** | **%** | **n** | **%** | **n** | **%** | **n** | **%** | **n** | **%** | **n** | **%** |
| 1-100 | 195 | 21.4 | 131 | 28.1 | 80 | 32.3 | 27 | 21.3 | 10 | 10.9 | 10 | 22.7 | 1 | 12.5 | 454 | 23.9 |
| 101-1,000 | 424 | 46.5 | 217 | 46.5 | 117 | 47.2 | 59 | 46.5 | 57 | 62.0 | 16 | 36.4 | 3 | 37.5 | 893 | 47.0 |
| 1,001-5,000 | 182 | 20.0 | 69 | 14.8 | 31 | 12.5 | 21 | 16.5 | 17 | 18.5 | 7 | 15.9 | 4 | 50.0 | 331 | 17.4 |
| 5,001-10,000 | 41 | 4.5 | 14 | 3.0 | 7 | 2.8 | 6 | 4.7 | 5 | 5.4 | 2 | 4.5 | 0 | 0.0 | 75 | 4.0 |
| 10,001-20,000 | 31 | 3.4 | 9 | 1.9 | 3 | 1.2 | 5 | 3.9 | 1 | 1.1 | 1 | 2.3 | 0 | 0.0 | 50 | 2.6 |
| 20,001-30,000 | 7 | 0.8 | 4 | 0.9 | 1 | 0.4 | 1 | 0.8 | 0 | 0.0 | 0 | 0.0 | 0 | 0.0 | 13 | 0.7 |
| >30,000 | 32 | 3.5 | 23 | 4.9 | 9 | 3.6 | 8 | 6.3 | 2 | 2.2 | 8 | 18.2 |  | 0.0 | 82 | 4.3 |
| Total | 912 | 100 | 467 | 100 | 248 | 100 | 127 | 100 | 92 | 100 | 44 | 100 | 8 | 100 | 1898 | 100 |

NPO: non-profit organization (non-profit, research institute, network).
†Exclude studies with n=0 (withdrawn).

Figure S1. Study flow diagram.

Clinical Trials Transformation Initiative

Aggregate Analysis of ClinicalTrials.gov database:

Studies identified by AI/ML search terms**†**

that started during 2010JAN1 to 2023DEC31

(n=3378)

272 studies excluded:

- 265 Not AI/ML-related (only ‘keyword’ hit with no AI/ML-terms used to describe research, or used but unrelated to study aim)‡
- 2 Systematic review/meta-analysis
- 5 ‘AI’ was part of org/product/study name

3106 AI/ML studies included

2264 not completed:

1694 Active

95 Stopped

475 Unknown

842 completed

Data downloaded on 6FEB2024 (i.e. last updated on 6FEB2024).

† Search strategy in Appendix Table 1.

‡ Examples: studies where AI/ML terms were used only in passing to merely refer to previous research or in forward-looking statements.

Figure S2. AI/ML terms used in study descriptions 2010-2023.

The figure shows terms used in official/brief titles, interventions, detailed descriptions, primary outcome, or keywords. Numbers can exceed the total number of studies as multiple techniques are often used within a study (4547 mentions in 3106 studies).

Figure S3. Clinical specialties Top 10 by start year, 2010-2023.

Figure S4. AI/ML terms in study descriptions by start year – Most frequently used Top 10 (n=3106).

Figure shows terms used in official/brief titles, interventions, conditions, detailed descriptions, and keywords. Numbers exceed the total number of studies as multiple techniques were often mentioned within a study (4547 mentions in 3106 studies).

**Figure S5.** Sample size by start year, 2010-2023.

Studies with n=zero (withdrawn) were excluded from interventional studies (n=1163) and observational studies (n=1898).

**Figure S6.** Sample size distributions compared across studies that also used ClinicalTrials.gov data.

Studies with n=zero (withdrawn) or n=unknown were excluded from interventional studies (n=1163) and observational studies (n=1898).

**Figure S7.** Studies for women over time.

| Start Year | All | Women | Men |
| --- | --- | --- | --- |
| 2010 | 12 | 0 | 0 |
| 2011 | 19 | 0 | 1 |
| 2012 | 18 | 1 | 0 |
| 2013 | 22 | 0 | 0 |
| 2014 | 20 | 2 | 0 |
| 2015 | 42 | 5 | 1 |
| 2016 | 61 | 3 | 0 |
| 2017 | 81 | 3 | 1 |
| 2018 | 187 | 15 | 4 |
| 2019 | 255 | 17 | 2 |
| 2020 | 438 | 18 | 6 |
| 2021 | 599 | 43 | 7 |
| 2022 | 672 | 52 | 12 |
| 2023 | 680 | 33 | 15 |
| Total | **3106** | **192** | **49** |

**Figure S8.** Lead sponsor sector - Original data with ‘OTHER’ versus Our reclassified data without ‘OTHER’

Academic hospitals were grouped under Hospital/Clinic.
